# The pretubulysin-induced exposure of collagen is caused by endothelial cell retraction that results in an increased adhesion and decreased transmigration of tumor cells

**DOI:** 10.18632/oncotarget.20746

**Published:** 2017-09-08

**Authors:** Rebecca Schwenk, Tanja Stehning, Iris Bischoff, Angelika Ullrich, Uli Kazmaier, Robert Fürst

**Affiliations:** ^1^ Institute of Pharmaceutical Biology, Goethe University, Frankfurt, Germany; ^2^ Institute of Organic Chemistry, Saarland University, Saarbrücken, Germany

**Keywords:** tumor cell adhesion, tumor cell transmigration, endothelium, extracellular matrix, pretubulysin

## Abstract

Microtubule-targeting agents (MTAs) are the most widely used chemotherapeutic drugs. Pretubulysin (PT), a biosynthetic precursor of the myxobacterial tubulysins, was recently identified as a novel MTA. Besides its strong anti-tumoral activities, PT attenuates tumor angiogenesis, exerts anti-vascular actions on tumor vessels and decreases cancer metastasis formation *in vivo*. The aim of the present study was to analyze the impact of PT on the interaction of endothelial and tumor cells *in vitro* to gain insights into the mechanism underlying its anti-metastatic effect. The influence of PT on tumor cell adhesion and transmigration onto/through the endothelium as well as its influence on cell adhesion molecules and the chemokine system CXCL12/CXCR4 was investigated. Treatment of human endothelial cells with PT increased the adhesion of breast cancer cells to the endothelial monolayer, whereas their transmigration through the endothelium was strongly reduced. Interestingly, the PT-induced upregulation of ICAM-1, VCAM-1 and CXCL12 were dispensable for the PT-evoked tumor cell adhesion. Tumor cells preferred to adhere to collagen exposed within PT-triggered endothelial gaps *via* β1-integrins on the tumor cell surface. Taken together, our study provides, at least in part, an explanation for the anti-metastatic potential of PT.

## INTRODUCTION

Microtubule-targeting agents (MTAs) are the most frequently used chemotherapeutic drugs. They can be classified into two main groups: the microtubule-stabilizing agents, such as taxanes (*e.g.* paclitaxel, docetaxel) or epothilones (*e.g.* ixabepilone), and the microtubule-destabilizing agents, such as vinca alkaloids (*e.g.* vincristine) or colchicine [1–3]. Microtubules are highly dynamic structures composed of continuously assembling and disassembling α,β-tubulin heterodimers (dynamic instability). They are present in all dividing and non-dividing cells and play an essential role in a wide range of cellular processes. The anti-cancer activity of MTAs was commonly attributed to their mitosis-blocking action, *i.e.* to their influence on the mitotic spindle apparatus. This view has changed during the last years: mitosis-independent actions on cancer cells, but also on other cell types, such as endothelial cells, have emerged as crucial anti-tumor mechanisms [1]. Due to the clinical success of the approved MTAs, but also because of their major drawbacks, such as resistance and side effects, the search for new classes of MTAs is still ongoing. In this context, in 2000, Sasse *et al.* described a novel group of highly potent microtubule-depolymerizing natural products referred to as tubulysins [4]. These compounds, which are produced by myxobacteria (*e.g. Archangium gephyra*), represent linear tetrapeptides that bind at the vinca domain of β-tubulin [4–7]. A crucial drawback of tubulysins is, as with other natural compounds, their sufficient supply. Fortunately, this obstacle has been overcome by the synthesis of simplified tubulysin derivatives [8–10], including pretubulysin (PT), a biosynthetic precursor of the tubulysins, which also acts as an MTA [11]. PT is chemically accessible and can be synthesized in the gram scale [12]. The chemical structures of PT and of the corresponding tubulysin D are depicted in Supplementary Figure 1. *In vitro*, PT was cytotoxic for different tumor cell lines (IC_50_: two-digit picomolar to low nanomolar range) [12]. Its strong anti-tumoral activities have been proven not only *in vitro*, but also *in vivo* [13–15]. Beyond tumor cells, PT was also reported to strongly influence endothelial cells: It attenuates tumor angiogenesis *in vivo* in a murine subcutaneous tumor model and in several *in vitro* test systems, such as endothelial migration or tube formation assays [14]. It also exerts profound anti-vascular actions on already existing tumor vessels *in vivo* in A-Mel-3 amelanotic melanoma tumors and *in vitro* on primary endothelial cells [16]. Interestingly, Braig *et al.* demonstrated that PT effectively decreases the formation of cancer metastases *in vivo* [15]. Hematogenous tumor metastasis is a multistep process: malignant cells from a primary tumor migrate and invade the surrounding tissue, intravasate into the vascular system and extravasate from blood vessels into distant organs, where they colonize to form secondary tumors [17]. Although only a few cancer cells of a primary tumor are able to form metastases [18, 19], tumor cell dissemination is one of the hallmarks of cancer and is responsible for 90 % of cancer-related human mortality [20]. Both the intra- and extravasation is based on the direct interaction of tumor cells with endothelial cells. The impact of PT on this interaction process has not been investigated so far. The aim of the present study was, therefore, to analyze its influence on the interaction of endothelial and tumor cells *in vitro* in order to gain insights into the mechanism underlying the anti-metastatic effect of PT. Beyond the known direct effect on tumor cells, we hypothesized that PT's anti-metastatic action is also based on alterations of endothelial cells.

## RESULTS

### Pretubulysin increases the adhesion and reduces the transmigration of tumor cells onto/through an endothelial monolayer

**Figure 1:**
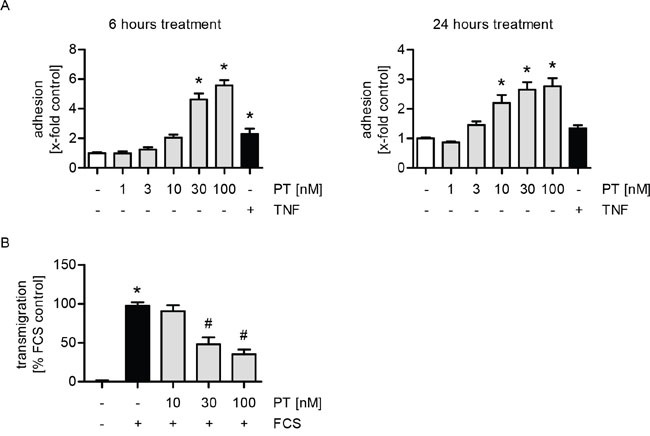
PT increases the adhesion and reduces the transmigration of MDA cells onto/through a HUVEC monolayer **(A)** Confluent HUVECs were treated with PT (1, 3, 10, 30, 100 nM) or TNFα (10 ng/ml) for 6 h (left) or 24 h (right). Fluorescence-labeled MDA cells were added and were allowed to adhere for 10 min. The amount of adherent MDA cells was determined by fluorescence measurements. **(B)** HUVECs were grown to confluence on a porous filter membrane (Transwell insert, polycarbonate membrane, 8 μm pores) and treated with PT (10, 30, 100 nM) for 24 h. Fluorescence-labeled MDA cells were added and were allowed to transmigrate for 24 h. The amount of transmigrated MDA cells on the lower part of the membrane was determined by fluorescence measurements. (A/B) Data are expressed as mean ± SEM. A left: n=3, A right: n=5, B: n=4. *p ≤0.05 versus negative control, #p ≤ 0.05 versus FCS control.

The adhesion of tumor cells onto the endothelium and their subsequent transendothelial migration represent two crucial steps in the metastatic process [17, 21]. We analyzed the influence of PT on HUVECs in *in vitro* cell adhesion and transmigration assays with MDA-MB-231 tumor cells. Of note, only the endothelial cells were treated with PT. We could show that treatment with PT for 6 or 24 h increases the adhesion of tumor cells onto the endothelial monolayer in a concentration-dependent manner (Figure 1A). TNFα, which is known to activate endothelial cells [22, 23], was used as a control. Interestingly, the transmigration of MDA-MB-231 cells trough the HUVEC monolayer was strongly reduced upon PT treatment in a concentration-dependent manner (Figure 1B).

### The enhanced expression of ICAM-1 and VCAM-1 is not linked to the PT-triggered tumor cell adhesion

**Figure 2:**
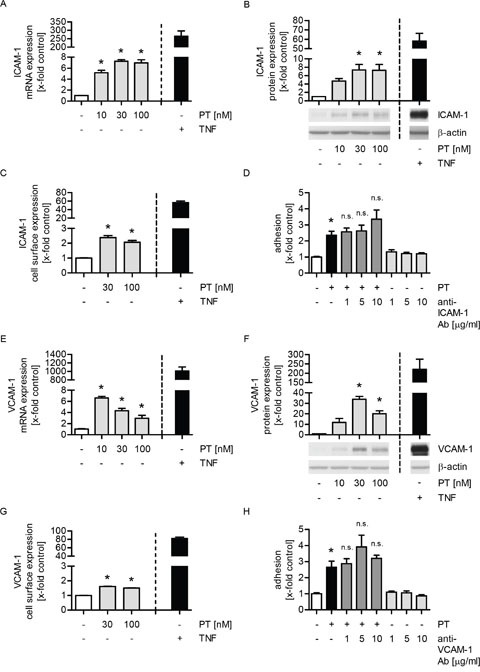
The enhanced expression of ICAM-1 or VCAM-1 is not responsible for the PT-triggered tumor cell adhesion **(A/E)** Confluent HUVECs were treated with PT (10, 30, 100 nM) or TNFα (10 ng/ml) for 12 h. The mRNA expression of ICAM-1 (A) or VCAM-1 (E) was analyzed by qPCR experiments. **(B/F)** Confluent HUVECs were treated with PT (10, 30, 100 nM) or TNFα (10 ng/ml) for 24 h. The total protein expression of ICAM-1 (B) or VCAM-1 (F) was determined by western blot analysis. **(C/G)** Confluent HUVECs were treated with PT (30, 100 nM) or TNFα (10 ng/ml) for 24 h. The cell surface expression of ICAM-1 (C) or VCAM-1 (G) was analyzed by flow cytometry. **(D/H)** Confluent HUVECs were treated with 30 nM PT for 24 h. An ICAM-1 blocking antibody (1, 5, 10 μg/ml) (D) or a VCAM-1 blocking antibody (1, 5, 10 μg/ml) (H) was added for the last 30 min of PT treatment. Fluorescence-labeled MDA cells were added and were allowed to adhere for 10 min. The amount of adherent MDA cells was determined by fluorescence measurements. (A-H) Data are expressed as mean ± SEM. A-C and E-H: n=3, D: n=5. *p ≤ 0.05 versus negative control, n.s.: p > 0.05 versus PT alone. TNFα was used as positive control and was not included into the statistical analyses.

We hypothesized that the increased adhesion of tumor cells onto the endothelium might be caused by an increased expression of endothelial adhesion molecules. The intercellular adhesion molecule 1 (ICAM-1), the vascular cell adhesion molecule 1 (VCAM-1), E-selectin and galectin-3 have been reported to be involved in the interaction of tumor and endothelial cells [24]. Regarding the influence of PT on the mRNA expression of these adhesion molecules, we found that PT treatment increased ICAM-1 (Figure 2A), VCAM-1 (Figure 2E) and E-selectin (Supplementary Figure 2A), but not galectin-3 levels (Supplementary Figure 2C). TNFα served as a positive control. Moreover, we studied the impact of PT on the total protein expression of ICAM-1 and VCAM-1 by western blot analysis. The total protein levels of ICAM-1 and VCAM-1 were clearly enhanced in PT-treated HUVECs (Figure 2B and 2F). The surface expression of ICAM-1 (Figure 2C) and VCAM-1 (Figure 2G) was increased by PT, whereas E-selectin (Supplementary Figure 2B) was not influenced. To prove whether the effect of PT on ICAM-1 or VCAM-1 expression plays a role in the PT-evoked tumor cell adhesion, we performed a cell adhesion assay, in which endothelial ICAM-1 or VCAM-1 was blocked after PT treatment by a neutralizing antibody before the (untreated) tumor cells were added. As shown in Figure 2D and 2H, the blocking of neither ICAM-1 nor VCAM-1 had any effect on the PT-induced tumor cell adhesion. These findings indicate that ICAM-1 and VCAM-1 do not participate in the PT-evoked adhesion of tumor cells to the endothelium.

### The PT-induced expression of CXCL12 is not linked to the increased tumor cell adhesion

**Figure 3:**
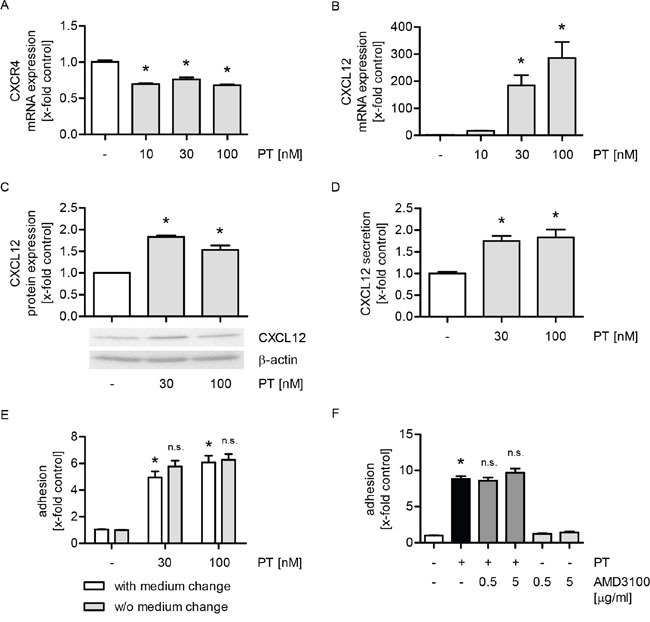
The increased expression of CXCL12 is not linked to the PT-evoked tumor cell adhesion **(A/B)** Confluent HUVECs were treated with PT (10, 30, 100 nM) for 12 h. The mRNA expression of CXCR4 (A) and CXCL12 (B) were analyzed by qPCR experiments. **(C/D)** Confluent HUVECs were treated with PT (30, 100 nM) for 24 h. (C) The total protein expression of CXCL12 was determined by western blot analysis. (D) CXCL12 levels in the cell supernatant were investigated by ELISA. **(E)** Confluent HUVECs were treated with PT (30, 100 nM) for 6 h. After treatment, the cell culture medium was removed and replaced by fresh medium (indicated as “with medium change”) or not (indicated as “w/o medium change”). **(F)** Confluent HUVECs were pretreated with the CXCR4 antagonist AMD3100 (0.5, 5 μg/ml) for 30 min. Then, PT (30 nM) was added for 24 h. (E/F) Fluorescence-labeled MDA cells were added and were allowed to adhere for 10 min. The amount of adherent MDA cells was determined by fluorescence measurements. (A-F) Data are expressed as mean ± SEM. A-D: n=3, E: n=5, F: n=4. *p ≤ 0.05 versus negative control, n.s.: p > 0.05 versus “with medium change” (E) or PT alone (F).

The chemokine system CXCL12/CXCR4 crucially regulates metastatic dissemination of breast cancer cells [25]. Hence, we were interested in the impact of PT on the expression of CXCR4 and, in particular, CXCL12 in HUVECs. The mRNA levels of CXCR4 were slightly reduced by PT (Figure 3A), whereas the mRNA expression of CXCL12 was strongly upregulated by PT in a concentration-dependent manner (Figure 3B). However, the total protein expression of CXCL12 (Figure 3C) and its secretion into cell culture supernatants (Figure 3D) were only slightly induced when HUVECs were treated with PT. CXCL12 secreted from endothelial cells might directly act on tumor cells. Thus, we performed a cell adhesion assay, in which the culture medium was either removed or not before MDA-MB-231 cells were added. As shown in Figure 3E, we observed no differences on the PT-triggered adhesion process. Thus, the secretion of CXCL12 (and cytokine secretion in general) is not obligatory for the PT-evoked tumor cell adhesion. To exclude a potential autocrine action of CXCL12 on HUVECs, we performed a cell adhesion assay, in which the CXCL12 receptor CXCR4 was inhibited with plerixafor (AMD3100) before MDA-MB-231 cells were added. AMD3100 did not affect the PT-induced tumor cell adhesion (Figure 3F). The functionality of AMD3100, i.e. its ability to block CXCR4 [26], was confirmed by western blot analysis (Supplementary Figure 3). Taken together, these data suggest that the chemokine system CXCL12/CXCR4 does not play any role in the action of PT.

### The PT-triggered increase of tumor cell adhesion is mediated by the exposure of extracellular collagen *via* endothelial cell retraction

**Figure 4:**
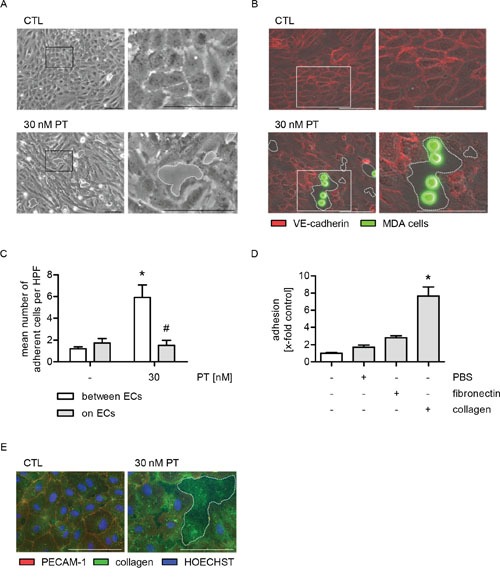
The PT-induced adhesion of MDA cells is based on the exposure of collagen in endothelial cell gaps **(A-C)** Confluent HUVECs were treated with 30 nM PT for 6 h. (A) The cells were washed with culture medium and phase-contrast images were obtained. (B) Fluorescence-labeled MDA cells (green) were added and were allowed to adhere for 10 min. Non-adherent cells were washed off. VE-cadherin (red) was visualized by immunocytochemistry and fluorescence microscopy. (C) The mean number of MDA cells per high-power field (HPF) that adhere either to endothelial gaps or onto ECs was determined by microscopic analysis and cell counting. **(D)** Fluorescence-labeled MDA cells were added to uncoated, PBS-treated, fibronectin- (5 μg/ml) or collagen-coated plastic (10 μg/ml) and were allowed to adhere for 10 min. The amount of adherent MDA cells was determined by fluorescence measurements. **(E)** Confluent HUVECs were treated with 30 nM PT for 6 h. PECAM-1 (red) and collagen (green) were visualized by immunocytochemistry and fluorescence microscopy. HOECHST was used for nuclei staining (blue). (C/D) Data are expressed as mean ± SEM. C: n=4, D: n=3. *p ≤ 0.05 versus negative control, #p ≤ 0.05 versus “30 nM PT within endothelial gaps”. Microscopic images: Dashed lines indicate interendothelial gaps. Scale bar represents 100 μm. One representative image out of at least 3 independently performed experiments is shown. A: n=3, B/E: n=4.

Recently, we have characterized PT as a vascular-disrupting compound [16] that leads to the formation of interendothelial gaps. The capability of PT to retract HUVECs was confirmed in Figure 4A (phase-contrast images; dashed lines indicate the gaps). Since the effects of PT are neither mediated by the adhesion molecules ICAM-1, VCAM-1, E-selectin and galectin-3, nor by the chemokine system CXCL12/CXCR4, we hypothesized that there might be an indirect interaction between HUVECs and MDA-MB-231 cells. Thus, we performed a cell adhesion assay, in which the endothelial cell boundaries and the tumor cells were visualized by immunofluorescence stainings of vascular endothelial cadherin (VE-cadherin, red) and by CellTracker green, respectively, in order to analyze the precise location of the tumor cells (Figure 4B). The microscopic images and the respective quantification (Figure 4B and 4C) clearly revealed that the tumor cells mainly adhere within the PT-triggered endothelial gaps (dashed lines).

Due to these findings, we assumed that the PT-triggered tumor cell adhesion might be based on an interaction between tumor cells and extracellular matrix proteins that are exposed within the PT-induced endothelial gaps. Consequently, we performed a cell adhesion assay and added MDA-MB-231 cells to PBS-, fibronectin- or collagen-coated plastic. The tumor cells showed a weak attachment to fibronectin, but strongly adhered to collagen (Figure 4D and Supplementary Figure 4). Immunofluorescence stainings revealed that collagen was present within the PT-induced endothelial gaps (Figure 4E, dashed line indicates the gap). Cell boundaries were visualized by immunofluorescence stainings of PECAM-1. Taken together, these data suggest that the PT-evoked tumor cell adhesion is based on the exposure of the extracellular matrix protein collagen.

### The effects of PT on tumor cell adhesion and transmigration are mediated by the interactions of β1-integrin on tumor cells with collagen within endothelial gaps

**Figure 5:**
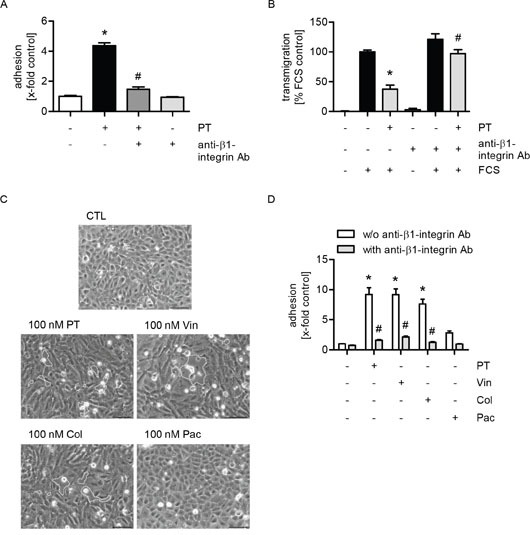
The effects of PT on tumor cell adhesion and transmigration are mediated by the interactions of β1-integrin on tumor cells with collagen within endothelial gaps **(A/D)** Confluent HUVECs were treated with 30 nM PT (A) or 100 nM PT (D) or vincristine, colchicine or paclitaxel (100 nM, each) for 6 h. Fluorescence-labeled MDA cells were left untreated or were incubated with a β1-integrin blocking antibody (1 μg/ml per 1 × 10^6^ cells) for 30 min. MDA cells were then added to the HUVEC monolayer and were allowed to adhere for 10 min. The amount of adherent MDA cells was determined by fluorescence measurements. **(B)** HUVECs were grown to confluence on a porous filter membrane (Transwell insert, polycarbonate membrane, 8 μm pores) and were treated with 100 nM PT for 24 h. Fluorescence-labeled MDA cells were left untreated or were incubated with a β1-integrin blocking antibody (1 μg/ml per 1 × 10^6^ cells) for 30 min. MDA cells were washed, added to the HUVEC monolayer and were then allowed to transmigrate through the endothelial monolayer for 24 h. The amount of transmigrated MDA cells on the lower part of the membrane was determined by fluorescence measurements. **(C)** Confluent HUVECs were treated with PT, vincristine, colchicine or paclitaxel (100 nM, each) for 6 h. The cells were washed with culture medium and phase-contrast images were obtained. (A/B; D) Data are expressed as mean ± SEM. A/D: n=3, B: n=5. *p ≤ 0.05 versus negative control (A/D) or FCS alone (B), #p ≤ 0.05 versus PT alone (A), PT + FCS (B) and “w/o anti-β1-integrin Ab” (D). Microscopic images: Dashed lines indicate interendothelial gaps. Scale bar represents 100 μm. One representative image out of 3 independently performed experiments is shown.

Since β1-integrins on tumor cells mediate interactions between cancer cells and extracellular matrix proteins in the gaps [27], their functional role was analyzed in cell adhesion and transmigration assays. β1-integrins on MDA-MB-231 cells were blocked by a neutralizing antibody before the tumor cells were added to the PT-treated HUVECs. We showed that both the PT-evoked increase in tumor cell adhesion (Figure 5A) and the decrease in transendothelial migration (Figure 5B) were completely abolished upon blockade of β1-integrins. The functionality of the used neutralizing antibody was confirmed by cell adhesion assays of MDA-MB-231 cells to collagen-coated plastic (Supplementary Figure 5). In summary, these results indicate that the action of PT might be based on the trapping of tumor cells within endothelial gaps due to the interaction of collagen with β1-integrins on tumor cells.

Moreover, to figure out whether the reported effects of PT depended on its depolymerizing action on the microtubule network, we analyzed the influence of other MTAs on endothelial cells. As with PT, treatment of endothelial cells with the microtubule-depolymerizing compounds vincristine and colchicine led to the formation of gaps within the endothelial monolayer (Figure 5C, dashed lines indicate gaps). In contrast, the microtubule-polymerizing substance paclitaxel did not affect the integrity of the endothelial monolayer (Figure 5C). Again, the role of β1-integrins on MDA-MB-231 cells was analyzed in cell adhesion assays: HUVECs were treated with the different MTAs. Besides PT, tumor cell adhesion was also induced by the treatment with vincristine and colchicine, whereas paclitaxel did only slightly affect this process (Figure 5D). Furthermore, we demonstrated that the compound-evoked increase in tumor cell adhesion was completely abolished when β1-integrins were blocked on the tumor cells (Figure 5D). Thus, we conclude that the observed effects of PT depend on its microtubule-depolymerizing activity.

## DISCUSSION

The influence of MTAs on endothelial cells is well studied regarding their anti-angiogenic and vascular-disrupting effects on the tumor microvasculature [28–31]. However, the impact of MTAs on endothelial cells of the *non-tumor* vasculature has been largely neglected. These cells play an essential role in metastasis, since they regulate the intra- and extravasation of hematogenically disseminated tumor cells [21, 32, 33]. In the present study, we analyzed the influence of PT on the interaction of non-tumor endothelial cells and breast cancer cells *in vitro*, since we hypothesized that PT influences the metastatic process – at least in part – *via* a direct action on the endothelium. We showed that the treatment of endothelial cells with PT strongly increased the adhesion of breast cancer cells to interendothelial gaps, but reduces their transmigration through the endothelial monolayer.

Various studies have focused on the interaction between tumor cells and endothelial cells of the non-tumor vasculature, investigating the regulatory mechanisms underlying tumor cell intra- and extravasation [34–38]. The adhesion molecules ICAM-1, VCAM-1, E-selectin and galectin-3 as well as the chemokine system CXCL12/CXCR4 have been reported to be involved in the interaction of tumor and endothelial cells. Thus, these molecules may play an essential role in cancer dissemination [24, 25]. Although the expression of ICAM-1 and VCAM-1 were significantly increased when endothelial cells were treated with PT, we demonstrated that this effect is not linked to the PT-evoked tumor cell adhesion. Also the CXCL12/CXCR4 system was not relevant for this action of PT. Interestingly, while the mRNA levels of CXCL12 were strongly increased in PT-treated endothelial cells, the secretion of CXCL12 from the endothelium was only marginally enhanced. Since PT interferes with microtubule dynamics resulting in the depolymerization of microtubules, it cannot be excluded that PT also affects microtubule-mediated transport processes. Thus, it might be possible that the CXCL12 mRNA is not successfully transported to the ribosomes for protein synthesis. We conclude that the effects of PT on the interaction of endothelial and tumor cells *in vitro* are based neither on an influence on cell adhesion molecules nor on the CXCL12/CXCR4 system.

We previously found that the treatment of endothelial cells with PT can lead to transient gaps within the endothelial monolayer, which are associated with the vascular-disruptive activity of the compound [16]. In accordance with these findings, instead of a direct interaction between endothelial and tumor cells, we observed that the tumor cells predominantly adhere to the extracellular matrix within interendothelial gaps. MDA-MB-231 cells only slightly adhered to fibronectin-coated surfaces, whereas they strongly stuck to collagen. Most importantly, we clearly showed that collagen was present within the PT-induced endothelial gaps.

Since the interaction between tumor cells and collagen is enabled by β1-integrins expressed on tumor cells [27], we examined their impact in cell adhesion and transmigration assays with MDA-MB-231 cells and PT-treated endothelial cells. Both the pretubulysin-evoked increase in cancer cell adhesion within endothelial gaps and the reduced transmigration through the endothelial monolayer were completely abolished when β1-integrins were blocked on the tumor cells. Besides PT, we observed the same effects also for the microtubule-depolymerizing compounds vincristine and colchicine. In contrast, the treatment with the microtubule-polymerizing agent paclitaxel did only very slighly affect this process. Thus, these results indicate that the observed effects of PT depend on its microtubule-depolymerizing activity [12–15].

In summary, our study showed that cell adhesion molecules and the CXCL12/CXCR4 chemokine system is not involved in the PT-induced interaction of tumor cells with the endothelium. PT triggers the exposure of the extracellular matrix component collagen by the formation of interendothelial gaps. As a consequence, tumor cells adhere to the exposed collagen, which leads to the inhibition of tumor cell transmigration. The results of our study provide, at least in part, an *in vitro* explanation for the anti-metastatic *in vivo* potential of the novel microtubule-targeting agent PT.

## MATERIALS AND METHODS

### Compounds

Pretubulysin was synthesized as described previously [12]. Colchicine (AG-CN2-0048), vincristine (AG-CN2-0446) and paclitaxel (AG-CN2-0045) were obtained from Biomol (Hamburg, Germany). Stock solutions (10 mM) were prepared in DMSO (Sigma-Aldrich, Taufkirchen, Germany) and stored at -20 °C. Substances were diluted in growth medium (concentrations as indicated) without exceeding a final DMSO concentration of 0.1 %. Recombinant human TNFα was obtained from PeproTech (Hamburg, Germany). CellTracker Green (C2925) was from Life Technologies (Darmstadt, Germany). Bovine serum albumin (BSA) was purchased from Sigma-Aldrich. Collagen G was obtained from Biochrom (Berlin, Germany) and fibronectin from Merck (Darmstadt, Germany). The anti-ICAM-1 antibody (15.2; sc-107) from Santa Cruz Biotechnology (Heidelberg, Germany), the anti-VCAM-1 monoclonal antibody (1.G11B1; MA5-16429) from (Thermo Fisher Scientific, Schwerte, Germany) and the anti-integrin β1 antibody (ab24693) from Abcam (Cambridge, UK) were used in blocking experiments. AMD3100 (plerixafor, sc-252367) was purchased from Santa Cruz Biotechnology.

### Cell culture

Human umbilical vein endothelial cells (HUVECs) were obtained from PELOBiotech (Martinsried, Germany), seeded in culture flasks or plates coated with collagen (10 μg/ml) in PBS and cultured in endothelial cell growth medium (ECGM, PELOBiotech) supplemented with 10 % fetal calf serum (FCS, Biochrom), 100 U/ml penicillin (PAN-Biotech, Aidenbach, Germany), 100 μg/ml streptomycin (PAN-Biotech) and 2.5 μg/ml amphotericin B (PAN-Biotech). MDA-MB-231 cells (MDA cells; ACC-732) were purchased from the Leibniz Institute for German Collection of Microorganisms and Cell Cultures (DSMZ, Braunschweig, Germany) and cultured in Dulbecco's Modified Eagle Medium (DMEM, PAN-Biotech) containing 10 % FCS, 100 U/ml penicillin and 100 μg/ml streptomycin. All cells and cell lines were cultured under constant humidity at 37 °C in an atmosphere of 5 % CO_2_. Phase contrast images were obtained by the inverted microscope Leica DM IL LED (Leica Microsystems).

### Adhesion of tumor cells to endothelial cells

HUVECs were grown to confluence in 24-well plates and treated as indicated in the respective figure legends. MDA-MB-231 cells were grown to confluence in a cell culture dish (10 cm) and were stained with CellTracker Green (5 μM) according to the manufacturer's instructions. MDA-MB-231 cells were resuspended in culture medium and were left untreated or treated with anti-β1-integrin antibody as described. Culture medium was removed from HUVECs, MDA-MB-231 cells (1 × 10^5^ cells per well) were added to the HUVEC monolayer and were allowed to adhere for 10 min. MDA-MB-231 cells that did not adhere were removed by washing three times with pre-warmed PBS. Fluorescence of adherent MDA-MB-231 cells was measured (ex: 485 nm; em: 535 nm) with a microplate reader (SPECTRAFluor Plus, Tecan, Männedorf, Switzerland) either directly in the culture plate or cells were lyzed with RIPA buffer (100 μl per well) before fluorescence measurements were performed.

### Adhesion of tumor cells to extracellular matrix proteins

24-well plates were coated with PBS, fibronectin or collagen for 2 h as described in the respective figure legends. MDA-MB-231 cells were stained with CellTracker Green as described above, resuspended in culture medium and left untreated or treated with anti-β1-integrin antibody as described. MDA-MB-231 cells (1 × 10^5^ cells per well) were added to the pre-coated wells and were allowed to adhere for 10 min. Non-adherent MDA-MB-231 cells were washed off and fluorescence of adherent MDA-MB-231 cells was measured as described above. For fluorescence microscopy, adhesion assays were performed in μ-slides (80826, ibidi, Martinsried, Germany) with MDA-MB-231 cells (5 × 10^4^ cells per well) as described above.

### Endothelial transmigration assay

Transwell inserts (diameter 6.5 mm, pore size 8 μm, polycarbonate membrane; Corning, New York, USA) were coated with collagen and HUVECs (1 × 10^5^ cells per insert) were seeded into the upper compartment according to the manufacturer's instructions. After 48 h, HUVECs were treated as indicated with PT. MDA-MB-231 cells were stained with CellTracker Green as described above, resuspended in medium 199 (PAN-Biotech) containing 0.1 % BSA and were left untreated or were treated with anti-integrin β1 antibody as indicated. Culture medium was removed from HUVECs and the cells were washed twice with medium 199 containing 0.1 % BSA. MDA-MB-231 cells (2 × 10^4^ cells per insert) were added on the upper compartment and were allowed to transmigrate through the endothelial monolayer for 24 h. In the lower compartment, medium 199 containing 0.1 % BSA was used for negative control, while medium 199 containing 20 % FCS served as positive control. Non-transmigrated cells on the upper compartment were carefully removed with a cotton swab. Transmigrated cells were lyzed by transferring the Transwell inserts into RIPA buffer (200 μl per insert). Fluorescence of transmigrated MDA-MB-231 cells was measured as described above.

### Quantitative polymerase chain reaction (qPCR)

HUVECs were grown to confluence in 6-well plates and treated with PT and TNFα as described. Total RNA was isolated with an RNeasy Mini Kit (Qiagen, Hilden, Germany) including on-column DNase digestion (RNase-Free DNase Set, Qiagen) according to the manufacturer's instructions. 1 μg of RNA was reversely transcribed using SuperScript II Reverse Transcriptase (Life Technologies). qPCR was performed with the StepOnePlus System (Applied Biosystems, Foster City, USA) based on the 2^-ΔΔCT^ method using the Power SYBR Green PCR Master Mix (Life Technologies). The following primers were used: ICAM-1 (forward, 5’-CTG CTC GGG GCT CTG TTC-3’; reverse, 5’-AAC AAC TTG GGC TGG TCA CA-3’), VCAM-1 (forward, 5’-CCA CAG TAA GGC AGG CTG TAA-3’; reverse, 5’-GCT GGA ACA GGT CAT GGT CA-3’), CXCR4 (forward, 5’-GCT GTT GGC TGA AAA GGT GG-3’; reverse, 5’-ATC TGC CTC ACT GAC GTT GG-3’), CXCL12 (forward, 5’-GAA AGC CAT GTT GCC AGA GC-3’; reverse, 5’-AGC TTC GGG TCA ATG CAC A-3’) and GAPDH (forward, 5’-CCA CAT CGC TCA GAC ACC AT-3’; reverse, 5’-TGA AGG GGT CAT TGA TGG CAA-3’). The threshold cycle for the gene of interest was normalized to that of *Gapdh*.

### Western blot analysis

HUVECs were grown to confluence in 6-well plates and treated as indicated with PT and TNFα as described. Cells were lyzed with RIPA buffer containing Complete Mini (Roche, Mannheim, Germany), sodium fluoride, phenylmethylsulphonyl fluoride and sodium vanadate. Protein concentrations were determined using the Pierce BCA Protein Assay Kit (Thermo Fisher Scientific). Proteins were separated by SDS polyacrylamide gel electrophoresis (Bio-Rad Laboratories, Munich, Germany) and transferred to polyvinylidene difluoride membranes by tank electroblotting (Bio-Rad Laboratories). Unspecific binding sites on the membranes were blocked with 5 % non-fat milk powder or BSA in PBS. Primary antibodies: mouse monoclonal anti-ICAM-1 antibody (15.2) (sc-107, 1:500), mouse monoclonal anti-VCAM-1 antibody (E-10) (sc-13160, 1:300) and mouse monoclonal anti-SDF-1 (CXCL12) antibody (sc-74271, 1:200) from Santa Cruz Biotechnology. Horseradish-peroxidase (HRP)-conjugated secondary antibodies: goat anti-mouse (sc-2005, 1:5000) antibody from Santa Cruz Biotechnology and mouse monoclonal anti-β-actin antibody (A3854, 1:100.000) from Sigma-Aldrich. Densitometric analysis was performed with the ImageJ software version 1.48v.

### Flow cytometric analysis

HUVECs were grown to confluence in 12-well plates and treated with PT and TNFα as described. Afterwards, HUVECs were washed twice with pre-warmed PBS and detached with HyClone HyQTase (GE Healthcare). In the case of ICAM-1, detached HUVECs were fixed with 4 % formaldehyde (Polysciences, Hirschberg an der Bergstraße, Germany) in PBS (final concentration: 2 %) and incubated with FITC-labeled anti-human CD54 (ICAM-1) antibody (MCA1615F, Biozol, Eching, Germany) in PBS. In the case of VCAM-1, detached HUVECs were left unfixed and incubated on ice with PE-labeled anti-human CD106 (VCAM-1) antibody (555647, Becton Dickinson, Heidelberg, Germany) in PBS containing 0.2 % BSA. Protein expression on the endothelial surface was analyzed by flow cytometry (FACSVerse, Becton Dickinson).

### Enzyme-linked immunosorbent assay (ELISA)

HUVECs were grown to confluence in 6-well plates and treated with PT as described. Cell supernatants were collected and cleared by centrifugation (13,300 rpm, 4 °C, 10 min). HUVECs were fixed with methanol/ethanol (2:1) and stained with crystal violet. Absorbance (540 nm) was measured with a microplate reader (Tecan). To determine CXCL12 levels in supernatants, the CXCL12/SDF-1 DuoSet ELISA (R&D Systems, Wiesbaden, Germany) was performed according to the manufacturer's instructions. Absorbance was measured at 450 nm and 540 nm and readings at 540 nm were subtracted from that at 450 nm to correct for optical imperfections in the plate. Data were finally normalized to that of crystal violet staining.

### Immunocytochemistry and fluorescence microscopy

HUVECs were grown to confluence on μ-slides coated with collagen and were treated with PT as indicated. Cells were directly fixed with Roti-Histofix (4 %, Carl Roth, Karlsruhe, Germany). For VE-cadherin analysis, cells were permeabilized with 0.2 % Triton X-100 (Sigma-Aldrich). Unspecific binding sites were blocked with 0.2 % BSA in PBS. Primary antibodies: mouse monoclonal anti-VE-cadherin (F-8) antibody (1:400, sc-9989, Santa Cruz Biotechnology), mouse monoclonal anti-PECAM-1 (JC70) antibody (1:400, sc-53411, Santa Cruz Biotechnology) and rabbit polyclonal anti-Collagen I + II + III + IV + V antibody (1:40, ab36064, Abcam). Secondary antibodies: Alexa Fluor 633-conjugated goat anti-mouse antibody (1:400, A21050) and Alexa Fluor 488-conjugated goat anti-rabbit antibody (1:400, A11008) from Life Technologies. HOECHST 33342 (1:10.000, 14533, Sigma-Aldrich) was used for nuclei staining. Images were obtained by a Leica DMI6000 B fluorescence microscope (Leica Microsystems, Wetzlar, Germany).

### Statistical analysis

Data were analyzed with the GraphPad Prism software version 5.0 (GraphPad Software, San Diego, USA). One-way ANOVA followed by Tukey's post-hoc test was used for the evaluation of three or more independently performed non-grouped experiments. Two-way ANOVA followed by Bonferroni post-hoc test was used for the evaluation of three or more independently performed grouped experiments. The numbers of performed experiments (n) are stated in the respective figure legends. P ≤ 0.05 was considered as statistically significant.

## SUPPLEMENTARY MATERIALS AND FIGURES



## Abbreviations

CXCL12: C-X-C motif chemokine 12
CXCR4: C-X-C chemokine receptor 4
ECM: Extracellular matrix
GAPDH: Glyceraldehyde 3-phosphate dehydrogenase
HUVEC: Human umbilical vein endothelial cell
ICAM-1: Intercellular adhesion molecule 1
MTA: Microtubule-targeting agent
PECAM-1: Platelet endothelial cell adhesion molecule 1
PT: Pretubulysin
SDF-1: Stromal cell-derived factor 1
TNFα: Tumor necrosis factor α
VCAM-1: Vascular cell adhesion molecule 1
VE-cadherin: Vascular endothelial cadherin
