# Supplementary material for: The pretubulysin-induced exposure of collagen is caused by endothelial cell retraction that results in an increased adhesion and decreased transmigration of tumor cells
Source: Oncotarget. 2017 Sep 8;8(44):77622–33. doi: 10.18632/oncotarget.20746 (PMC5652804; doi:10.18632/oncotarget.20746)
Supplement: Supplementary file 1 [file oncotarget-08-77622-s001.pdf]

The pretubulysin-induced exposure of collagen is caused by endothelial cell retraction that results in an increased adhesion and decreased transmigration of tumor cells

SUPPLEMENTARY MATERIALS

Quantitative polymerase chain reaction (qPCR)

qPCR experiments were performed as described in the main article. Additionally, the following primers were used: E-selectin (forward, 5'-AGA TGA GGA CTG CGT GGA GA-3'; reverse, 5'-GTG GCC ACT GCA GGA TGT AT-3') and galectin-3 (forward, 5'-GCC TTC CAC TTT AAC CCA CG-3'; reverse, 5'-ACT GCA ACC TTG AAG TGG TCA-3').

Flow cytometric analysis

Flow cytometric analysis was performed as described in the main article. As with VCAM-1, detached HUVECs were left unfixed and incubated on ice. The following antibody was used: PE-labeled anti-human CD62E (E-selectin) antibody (551145, Becton Dickinson).

Western blot analysis

Western blot analysis was performed as described in the main article. Recombinant human SDF-1α (CXCL12) was obtained from PeproTech. The following antibodies were used: mouse monoclonal anti-phospho-p44/42 (Erk1/2) (E-10) antibody (9106, 1:2000) and rabbit polyclonal anti-p44/42 (Erk1/2) antibody (9102, 1:1000) from Cell Signaling Technology (Leiden, Netherlands) and (HRP)-conjugated goat anti-rabbit antibody (sc-2004, 1:1000) from Santa Cruz Biotechnology.

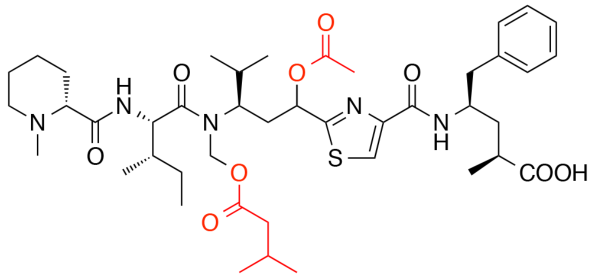

tubulysin D

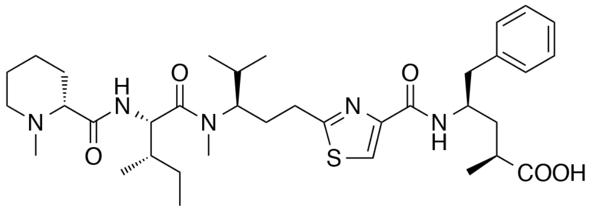

pretubulysin

Supplementary Figure 1: Chemical structure of tubulysin D and the corresponding pretubulysin. Differences are marked in red.

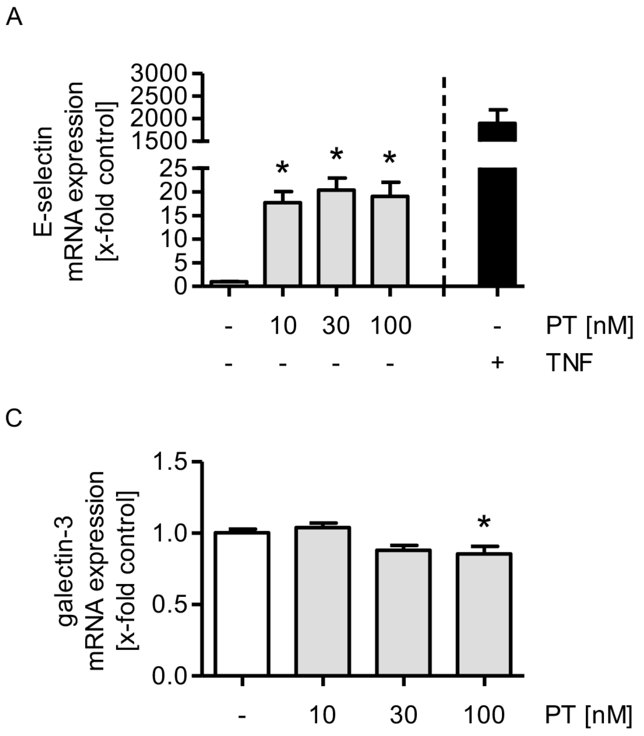

Supplementary Figure 2: PT does not influence the surface expression of E-selectin and the mRNA expression of galectin-3. (A/C) Confluent HUVECs were treated with PT (10, 30, 100 nM) or TNFα (10 ng/ml) (A) for 12 h. The expression of E-selectin (A) and galectin-3 (C) was analyzed on mRNA level by qPCR experiments. **(B)** Confluent HUVECs were treated with PT (30, 100 nM) or TNFα (10 ng/ml) for 4 h. The cell surface expression of E-selectin was analyzed by flow cytometry. Data are expressed as mean ± SEM (n=3). $^*p \leq 0.05$ versus control.

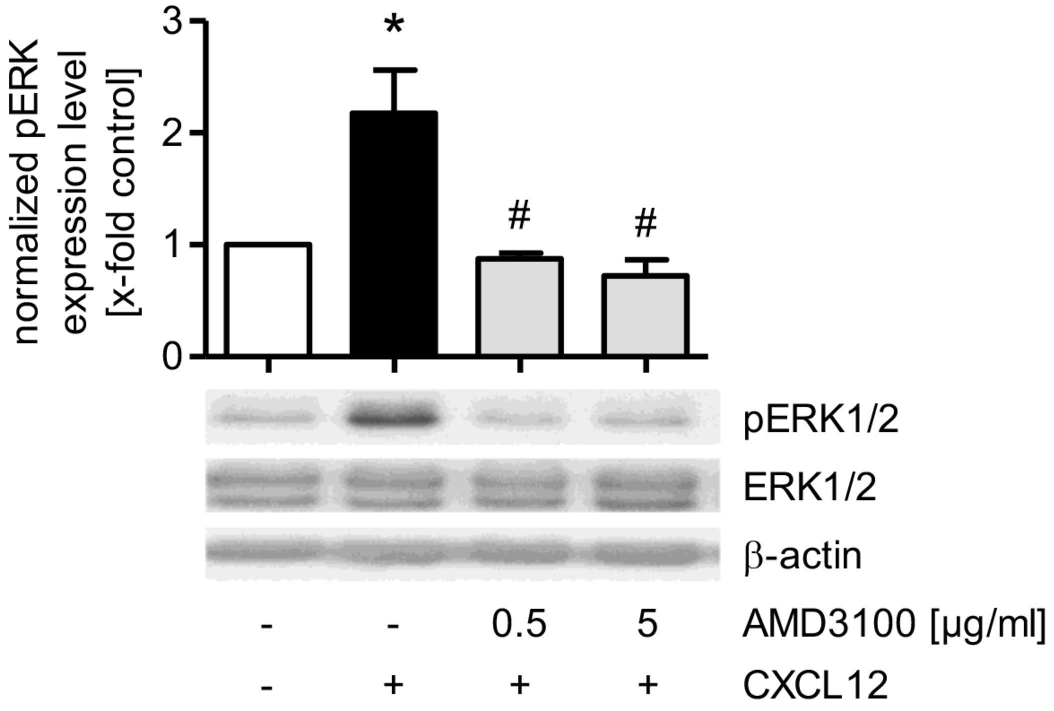

Supplementary Figure 3: Proof of the functionality of the CXCR4 inhibitor AMD3100. Confluent HUVECs were treated with AMD3100 (0.5 or 5 µg/ml) for 30 min. CXCL12 (500 ng/ml) was added for the last 5 minutes of AMD3100 treatment. The protein expression of phosphorylated ERK1/2 (pERK1/2) and ERK1/2 was determined by western blot analysis. Levels of ERK1/2 were normalized to β-actin and, subsequently, levels of pERK1/2 were normalized to ERK1/2. Data are expressed as mean ± SEM (n=3). *$p \leq 0.05$ versus control, $^{\#}p \leq 0.05$ versus CXCL12 alone.

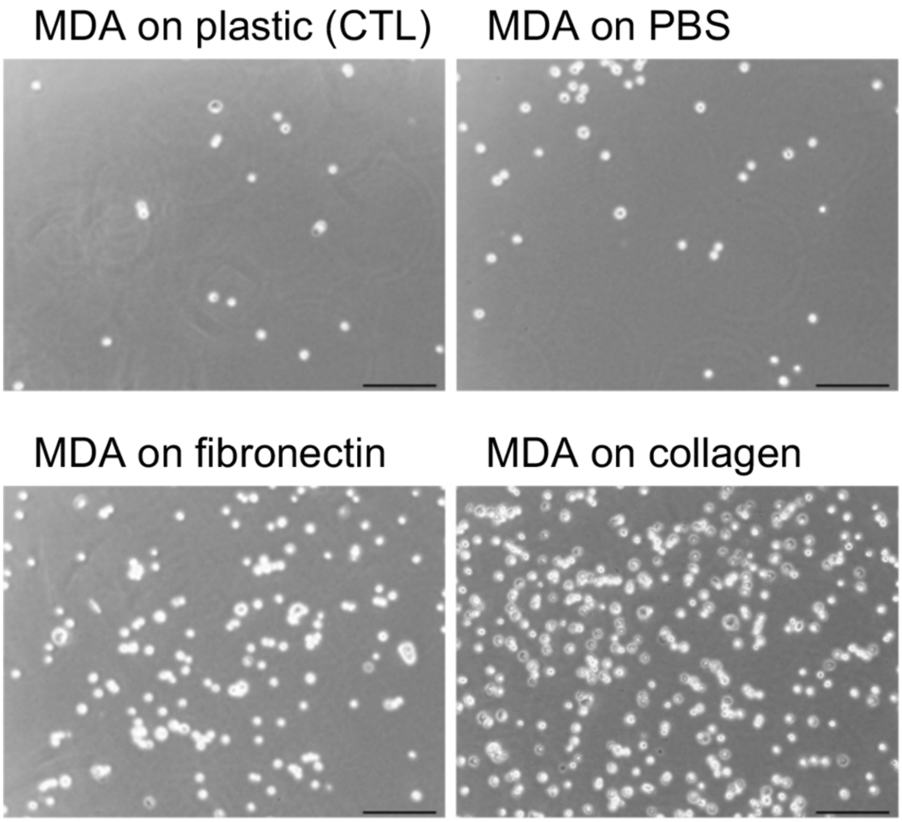

Supplementary Figure 4: Microscopical images corresponding to Figure 4D. Fluorescence-labeled MDA cells were added to uncoated, PBS-treated, fibronectin- (5 µg/ml) or collagen-coated plastic (10 µg/ml) and were allowed to adhere for 10 min. The cells were washed with culture medium and phase-contrast images were obtained. Microscopical images: Scale bar represents 100 µm. One representative image out of 3 independently performed experiments is shown.

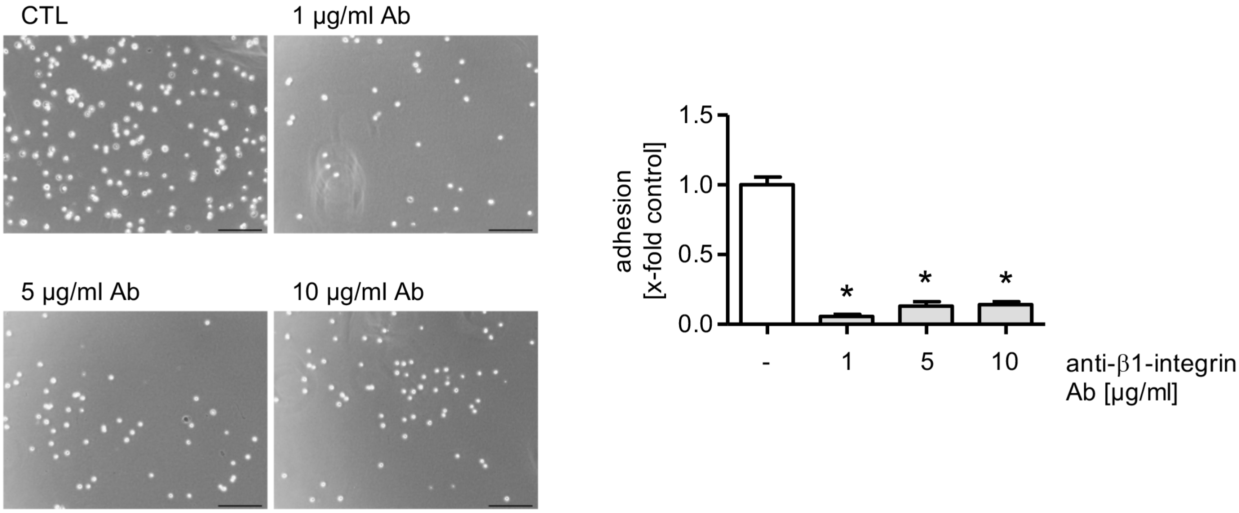

Supplementary Figure 5: Proof of the functionality of the anti-β1-integrin antibody. Fluorescence-labeled MDA cells were left untreated or were incubated with an anti-β1-integrin blocking antibody (1, 5, 10 µg/ml per 1 x 10^6 cells) for 30 min. MDA cells were then added to collagen-coated (10 µg/ml) wells and were allowed to adhere for 10 min. The cells were washed with culture medium and phase-contrast images were obtained (left) or the amount of adherent MDA cells was determined by fluorescence measurements (right). Data are expressed as mean ± SEM (n=3). *$p \leq 0.05$ versus control. Microscopical images: Scale bar represents 100 µm. One representative image out of 3 independently performed experiments is shown.
